# Percutaneous Endoscopic Posterior Lumbar Interbody Fusion with Unilateral Laminotomy for Bilateral Decompression Vs. Open Posterior Lumbar Interbody Fusion for the Treatment of Lumbar Spondylolisthesis

**DOI:** 10.3389/fsurg.2022.915522

**Published:** 2022-05-25

**Authors:** Li-Ming He, Jia-Rui Li, Hao-Ran Wu, Qiang Chang, Xiao-Ming Guan, Zhuo Ma, Hao-Yu Feng

**Affiliations:** ^1^ Department of Orthopaedic Surgery, Shanxi Bethune Hospital, Shanxi Academy of Medical Sciences, Taiyuan, China; ^2^ Department of Orthopaedic Surgery, Third Hospital of Shanxi Medical University, Taiyuan, Shanxi, China; ^3^ Department of Orthopaedic Surgery, Tongji Shanxi Hospital, Taiyuan, China

**Keywords:** unilateral laminotomy for bilateral decompression, percutaneous endoscopy, posterior lumbar interbody fusion, lumbar spondylolisthesis, lumbar spinal stenosis

## Abstract

**Background:**

Endoscopic lumbar interbody fusion is a new technology that is mostly used for single-segment and unilateral lumbar spine surgery. The purpose of this study is to introduce percutaneous endoscopic posterior lumbar interbody fusion (PE-PLIF) with unilateral laminotomy for bilateral decompression (ULBD) for lumbar spondylolisthesis and evaluate the efficacy by comparing it with open posterior lumbar interbody fusion (PLIF).

**Methods:**

Twenty-eight patients were enrolled in PE-PLIF with the ULBD group and the open PLIF group. The perioperative data of the two groups were compared to evaluate the safety of PE-PLIF with ULBD. The visual analog scale (VAS) back pain, VAS leg pain, and Oswestry Disability Index (ODI) scores of the two groups preoperatively and postoperatively were compared to evaluate clinical efficacy. Preoperative and postoperative imaging data were collected to evaluate the effectiveness of the operation.

**Results:**

No differences in baseline data were found between the two groups (*p* > 0.05). The operation time in PE-PLIF with the ULBD group (221.2 ± 32.9 min) was significantly longer than that in the PLIF group (138.4 ± 25.7 min) (*p* < 0.05), and the estimated blood loss and postoperative hospitalization were lower than those of the PLIF group (*p* < 0.05). The postoperative VAS and ODI scores were significantly improved in both groups (*p* < 0.05), but the postoperative VAS back pain score in the PE-PLIF group was significantly lower than that in the PLIF group (*p* < 0.05). The excellent and good rates in both groups were 96.4% according to MacNab’s criteria. The disc height and cross-sectional area of the spinal canal were significantly improved in the two groups after surgery (*p* < 0.05), with no difference between the groups (*p* > 0.05). The fusion rates in PE-PLIF with the ULBD group and the PLIF group were 89.3% and 92.9% (*p* > 0.05), respectively, the cage subsidence rates were 14.3% and 17.9% (*p* > 0.05), respectively, and the lumbar spondylolisthesis reduction rates were 92.72 ± 6.39% and 93.54 ± 5.21%, respectively (*p* > 0.05).

**Conclusion:**

The results from this study indicate that ULBD can be successfully performed during PE-PLIF, and the combined procedure is a safe and reliable treatment method for lumbar spondylolisthesis.

## Introduction

Joson and McCormick (1) reported a unilateral approach for bilateral decompression with preservation of the supraspinous ligament complex. Poletti (2) initially utilized unilateral laminotomy for bilateral ligamentectomy for lumbar stenosis caused by a thickened ligamentum flavum by establishing a working area through the excision of the ipsilateral laminae and spinous process roots, followed by partial excision of the contralateral lamina and ligamentum flavum to decompress the spinal canal. Spetzger et al. (3) first proposed the concept of unilateral laminotomy for bilateral decompression (ULBD). With advancements in technology, surgeons introduced tubular technology and endoscopic technology into ULBD, achieving satisfactory clinical outcomes (4–7).

Endoscopic lumbar interbody fusion is a new technology and a research hotspot with many advantages, such as significant improvement in surgical visualization and enhanced recovery after surgery (8). We performed percutaneous endoscopic posterior lumbar interbody fusion (PE-PLIF) in 2019. PE-PLIF is a uniportal endoscopic technique with the working channel established through the excision of the medial part of the facet joint and part of the ipsilateral lamina. This methodology has been shown to be a safe and effective method in our preliminary studies (9).

However, for patients with lumbar spondylolisthesis complicated by neurological symptoms in both lower extremities or intermittent claudication, the unilateral approach of PE-PLIF is not suitable, and the bilateral PE-PLIF will obviously increase surgical trauma and operative time in our experience. Therefore, we combined PE-PLIF with ULBD to treat such patients. This report discusses the differences between ULBD procedures in PE-PLIF and classical ULBD procedures and evaluates the safety and efficacy of PE-PLIF with ULBD by comparing it with open PLIF.

## Materials and Methods

### Study Design

This study was approved by the Ethics Committee of Shanxi Bethune Hospital, and written permission was obtained from all included patients. This study was a retrospective study using the guidelines of Strengthening the Reporting of Observational Studies in Epidemiology (STROBE) (10). All surgeries were performed by a team of surgeons. The inclusion criteria were as follows: (1) single-segment lumbar spondylolisthesis (Meyerding grades I and II) with lumbar spinal stenosis; (2) conservative treatment was ineffective for more than 3 months, or symptoms were progressively aggravated; and (3) an age over 18 years. The exclusion criteria were as follows: (1) multisegment lumbar degenerative disease shown by imaging examination and (2) spinal deformities, old fractures, ankylosing spondylitis, or rheumatoid arthritis. For convenience, “the PE-PLIF group” in the text denotes PE-PLIF with ULBD.

### Surgical Techniques

**Figure 1:**
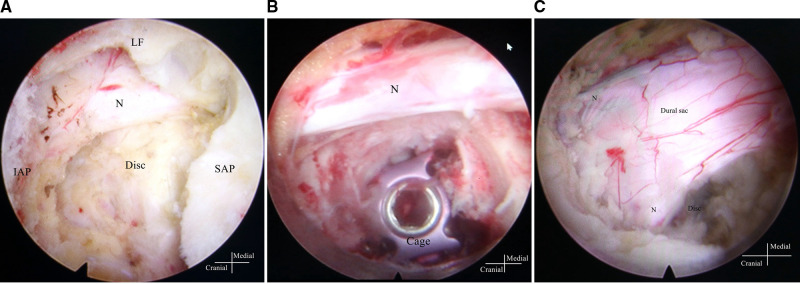
Images under endoscopy. (**A**) The SAP is exposed after the IAP is excised, and the nerve root and disc are exposed after the SAP is excised. (**B**) The cage and the nerve root after inserting the cage. (**C**) The dural sac and the bilateral nerve root after unilateral laminotomy for bilateral decompression. SAP, superior articular process; IAP, inferior articular process; LF, ligamentum flavum; N, nerve root.

**Figure 2:**
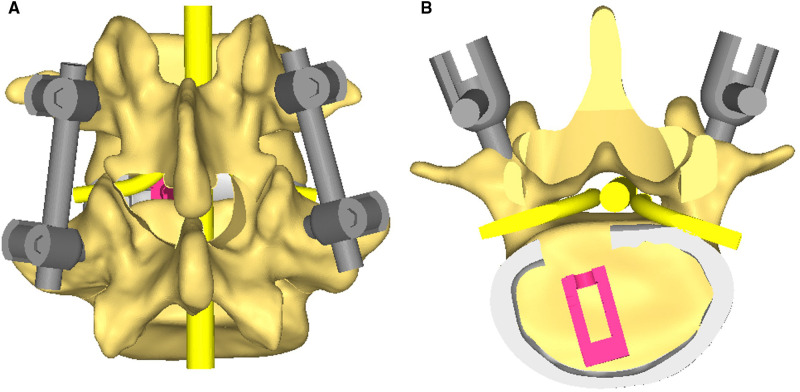
(**A**) A 3D schematic diagram of percutaneous endoscopic posterior lumbar interbody fusion with unilateral laminotomy for bilateral decompression. (**B**) A cross-sectional schematic diagram.

**Figure 3:**
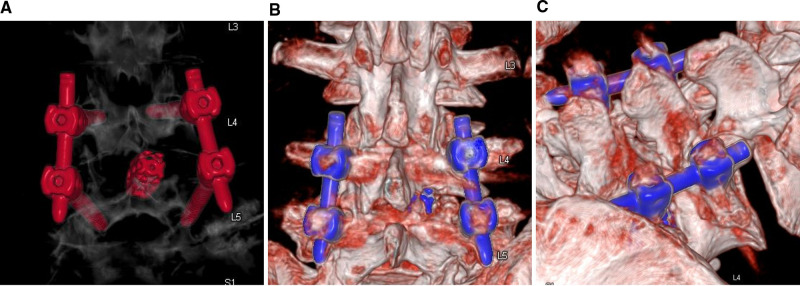
CT reconstructed images. (**A,B**) The extent of intraoperative laminectomy and facetectomy. (**C**) The base of the spinous process is excised.

PE-PLIF with ULBD: PE-PLIF has been described in detail in previous reports (9). The briefly described procedures are as follows: The patient is placed in the prone position after the induction of general anesthesia. The insertion point is marked at approximately 2 cm from the midline under anteroposterior X-ray. A longitudinal incision of approximately 13 mm is created after positioning the insertion point. After gradually expanding the soft tissues, a working sleeve (11-mm inner diameter) and an endoscope are placed (LUSTA endoscope system, Spinendos, Germany, a 10-mm outer diameter, 7.1-mm working channel, and 15° view angle). The medial portion of the articular process is excised until the working tube can be safely accommodated (Figure 1A). A part of the ligamentum flavum is excised to expose the nerve roots, the dural sac, and the intervertebral disc. The nerve roots are protected, discectomy is performed, and endplates are placed. The endoscopy is removed and a funnel-shaped bone graft device is inserted. After grafting the bone into the intervertebral space, an expandable interbody fusion cage is placed and expanded to a suitable height (9–13 mm) under a C-arm (Figure 1B). In this procedure, the bevel of the cannula is toward the lateral side to prevent the nerve roots from entering the working space. ULBD is performed as detailed in previous reports (5, 11), with a brief description provided as follows: A grinding drill and a lamina forceps are used to excise the margin of the ipsilateral superior lamina until the superior limit of the ligamentum flavum attachment and the margin of the ipsilateral inferior lamina. The ipsilateral ligamentum flavum is excised. The base of the spinous process is sawed off to expose the contralateral ligamentum flavum and lamina. The contralateral lamina and ligamentum flavum are excised in the same manner. Finally, a part of the contralateral articular process is excised to expose the contralateral nerve root, and decompression is performed (Figure 1C). After endoscopic examination of the decompression and fusion cage location, bilateral percutaneous pedicle screw internal fixation is performed. Figure 2 shows a schematic diagram of PE-PLIF with ULBD and Figure 3 shows a postoperative CT reconstruction image.

Open PLIF: The patient is placed in the prone position after the induction of general anesthesia. The operative segment is determined under a C-arm. A posterior median incision of approximately 8 cm is created. The paraspinal muscles are split to expose the lamina and bilateral facet joints. Complete laminar decompression is performed. The medial parts of the superior and inferior facet joints are excised to expose the nerve roots. The nerve roots and dural sac are protected, the intervertebral disc is excised, and endplates are prepared. After testing the model, a conventional cage filled with autologous bone and osteoinductive materials is placed into the intervertebral space. Pedicle screw internal fixation is performed on the operative segment.

### Clinical Evaluation

Perioperative data: Operation time: the time between needle positioning and skin suture. Estimated blood loss: Intraoperative blood loss plus the postoperative drainage volume. If the patient has cerebrospinal fluid leakage, the bleeding volume can be estimated by stratifying the drainage fluid. Complications: Surgery-related complications occurring during the operation or within 1 month after the operation. Postoperative hospital stay: The number of days between the day of surgery and the day of discharge.

Clinical results: VAS scores (0–10) for back pain and leg pain were recorded before surgery, 1 week after surgery, 1 month after surgery, 6 months after surgery, and at the last follow-up. The ODI score (0–100) was recorded to evaluate functional status before surgery, 1 month after surgery, 6 months after surgery, and at the last follow-up. Patient satisfaction rates were calculated according to the MacNab criteria (12). All questionnaires were completed by a doctor during an appointment or via telephone. At the same time, a quality controller was set up to evaluate the quality of the questionnaire.

### Imaging Evaluation

Preoperative and postoperative imaging data were measured and are listed below. Lumbar lordotic angle (LLA): the angle between the parallel line of the superior end plate of the lumbar 1 vertebra and the parallel line of the superior end plate of the sacrum. Segmental lordotic angle (SLA): L4–L5 is the angle between the parallel line of the upper end plate of L4 and the parallel line of the lower end plate of L5, and L5–S1 is the angle between the upper end plate of L5 and the upper end plate of S1. Disc height (DH): The average value of the distance from the upper endplate to the lower endplate. Cross-sectional area of the spinal canal (CSAC): The area of the spinal canal is measured on T2WI axial images. The front is bounded by the intervertebral disc, the back is bounded by the anterior border of the ligamentum flavum, and the two sides are bordered by the outer border of the nerve root. The above parameters were measured according to the study of Lin et al. (13). Reduction rate of lumbar spondylolisthesis (RLS): (the relative displacement distance of vertebral body on preoperative lateral X-ray – the relative displacement distance of vertebral body on postoperative lateral X-ray) / the relative displacement distance of the vertebral bodies on preoperative lateral X-ray. These distances were measured using the techniques described by Posner et al. (14) and Dupuis et al. (15). Fusion evaluation: The Birdwell criteria (16) were used to evaluate the X-ray or CT images at the last follow-up. Cage subsidence was defined as a cage entering the endplate by more than 2 mm (13). The LLA, SLA, DH, or CSAC changes were calculated as the postoperative data minus the preoperative data. All imaging measurements were performed on the picture archives communication system, syngo.plaza (Siemens, Germany). All data were evaluated by two senior spine surgeons who were blinded to the situation.

### Statistical Analysis

The data are displayed as the mean ± standard deviation. Continuous variables such as age, VAS score, ODI score, SLA, LLA, and DH were analyzed with the independent sample *t*-test for intergroup comparisons and the paired *t*-test for intragroup comparisons. Nominal data, such as segment, satisfaction rate, and fusion rate, were analyzed with the *χ*^2^ test or Fisher’s exact test. A *p*-value <0.05 was considered indicative of statistical significance. Statistical analysis was performed using IBM SPSS, version 26.0 (IBM Corp., Armonk, NY, USA).

## Results

### Demographic Data

Fifty-six patients between January 2020 and August 2020 were included according to the inclusion and exclusion criteria, with 28 cases in the PE-PLIF group, an average age of 59.8 ± 10.9 years (31–78 years), 14 males and 14 females, 20 cases at the L4–L5 segment, and 8 cases at the L5–S1 segment. In addition, 28 patients in the PLIF group were included as the control group: the average age was 54.2 ± 10.3 years (31–74 years), with 13 males and 15 females, 17 cases at the L4–L5 segment, and 11 cases at the L5–S1 segment. Detailed demographic data are given in Table 1. No significant difference was found in the baseline characteristic data between the PE-PLIF and the open PLIF groups (*p* > 0.05) (Table 1).

**Table l:** Comparison of demographic data and perioperative data.

	PE-PLIF (*n* = 28)	Open PLIF (*n* = 28)	*p*
Age (years)	59.8 ± 10.9	54.2 ± 10.3	0.053
Sex ratio (male/female)	14/14	13/15	0.789^a^
BMI	24.6 ± 2.0	24.4 ± 3.5	0.851
Smoke (yes/no)	11/17	8/20	0.573^a^
Diabetes mellitus (yes/no)	2/26	3/25	1.000^a^
Osteoporosis (yes/no)	2/26	2/26	1.000^a^
Segment (L4–L5/L5–S1)	20/8	17/11	0.397^a^
Meyerding grade (I/II)	11/17	13/15	0.787^a^
Mean follow-up (months)	18.4 ± 1.3	18.9 ± 1.7	0.161
Operative times (min)	221.2 ± 32.9	138.4 ± 25.7	<0.001
ULBD time (min)	33.3 ± 6.7		
Estimated blood loss (ml)	169.2 ± 49.5	649.6 ± 119.9	<0.001
Postoperative hospitalization (days)	3.5 ± 0.6	7.3 ± 1.5	<0.001

*BMI, body mass index; PE-PLIF, percutaneous endoscopic posterior lumbar interbody fusion; Open PLIF, open posterior lumbar interbody fusion.*

^a^ *Results from Fisher’s exact test or χ^2^ test.*

### Perioperative Outcomes

The operative time in the PE-PLIF group was significantly longer than that in the PLIF group (*p* < 0.05), with an average of 33.3 ± 6.7 min for the ULBD procedure. The estimated blood loss and postoperative hospitalization rate in the PE-PLIF group were significantly lower than those in the PLIF group (*p* < 0.05) (Table 1). One patient in the PE-PLIF group experienced a dural tear, and the drainage tube was removed the day after the operation. The patient did not have any related symptoms. One patient in the PLIF group experienced a dural tear, and the drainage tube was removed 10 days after surgery when the volume of drainage was significantly reduced.

### Clinical Efficacy

No significant differences in preoperative scores were identified between the two groups (*p* > 0.05). Both groups had significantly improved postoperative VAS back pain, VSA leg pain, and ODI scores (*p* < 0.05). The VAS back pain score in the PE-PLIF group was lower than that in the PLIF group at each postoperative time point (*p* < 0.05). No significant difference in the VAS leg pain score was noted between the two groups at any postoperative time point (*p* > 0.05). One month after the operation, the ODI score in the PE-PLIF group was lower than that in the PLIF group. No significant difference in the ODI score was found between the two groups at other postoperative time points (*p* > 0.05) (Table 2). The above results indicated that the lower back pain score in the PE-PLIF group was lower than that in the PLIF group, and the PE-PLIF group recovered faster than the PLIF group. According to the MacNab criteria, the PE-PLIF group had excellent outcomes in 20 cases, good outcomes in 7 cases, and a fair outcome in 1 case. PLIF group: excellent outcomes in 21 cases, good outcomes in 6 cases, and a fair outcome in 1 case. The excellent and good rates in both groups were 96.4%.

**Table 2:** The clinical outcomes of the two groups.

	PE-PLIF (*n* = 28)	Open PLIF (*n* = 28)	*p*
VAS back pain			
Preoperation	4.61 ± 1.42	4.64 ± 1.39	0.925
Postoperation			
1 week	2.25 ± 0.65*	3.21 ± 0.42*	<0.001
1 month	1.46 ± 0.58*	2.04 ± 0.51*	<0.001
6 months	0.79 ± 0.57*	1.14 ± 0.52*	0.018
Last	0.64 ± 0.49*	1.14 ± 0.36*	<0.001
VAS leg pain			
Preoperation	6.29 ± 0.85	6.21 ± 0.88	0.759
Postoperation			
1 week	2.46 ± 0.74*	2.32 ± 0.55*	0.417
1 month	1.21 ± 0.50*	1.36 ± 0.49*	0.283
6 months	0.71 ± 0.60*	0.89 ± 0.42*	0.201
Last	0.68 ± 0.55*	0.61 ± 0.50*	0.612
ODI			
Preoperation	47.36 ± 5.31	45.61 ± 3.87	0.841
Postoperation			
1 month	22.89 ± 4.24*	29.82 ± 5.32*	<0.001
6 months	12.61 ± 3.54*	12.79 ± 3.37*	0.847
Last	10.68 ± 2.86*	9.29 ± 3.22*	0.092

*VAS, visual analog scale; ODI, Oswestry Disability Index; PE-PLIF, percutaneous endoscopic posterior lumbar interbody fusion; Open PLIF, open posterior lumbar interbody fusion*.

* *p < 0.05 compared with the preoperative data*.

### Radiographic Parameters

No significant differences in preoperative radiographic parameters were identified between the two groups (*p* > 0.05), except for the LLA (*p* < 0.05). Since a difference in the LLA was found between the two groups, LLA changes were compared to evaluate the difference between the two groups (*p* > 0.05). Both groups did not significantly improve the LLA or SLA after surgery (*p* > 0.05). Both groups had significantly improved DHs, and a partial loss of the DH was observed at the last follow-up. The postoperative CSAC was significantly improved in both groups (*p* < 0.05), and the postoperative CSAC in the PLIF group was slightly larger than that in the PLIF group, although with no significant difference (*p* > 0.05). No significant differences in the SLA, DH, or CSAC changes were identified between the two groups (*p* > 0.05). The RLSs were 92.72 ± 6.39% with PE-PLIF and 93.54 ± 5.21% with PLIF, with no significant differences between the two groups (*p* < 0.05). The interbody fusion rate in the PE-PLIF group was 89.3% (Birdwell I 25, II 3), and the rate in the PLIF group was 92.9% (Birdwell I 26, II 2). The incidence rates of fusion device settlement were 14.3% (4/28) in the PE-PLIF group and 17.9% (5/28) in the PLIF group. No significant differences in the fusion rate or cage subsidence were noted between the two groups (*p* > 0.05) (Table 3).

**Table 3:** The radiographic outcomes in the PE-PLIF and open PLIF groups.

	PE-PLIF	Open PLIF	*p*
LLA (°)			
Preoperation	35.36 ± 10.27	40.93 ± 7.09	0.022
Postoperation	38.50 ± 7.68	41.75 ± 6.11	0.085
LLA change (°)	0.68 ± 2.04	0.64 ± 3.50	0.963
SLA (°)			
Preoperation	15.86 ± 4.37	17.18 ± 3.39	0.211
Postoperation	15.64 ± 3.42	17.21 ± 3.02	0.074
SLA change (°)	−0.21 ± 2.13	−0.11 ± 2.27	0.856
DH (mm)			
Preoperation	8.66 ± 1.45	8.75 ± 1.65	0.844
Postoperation	11.42 ± 1.19*	11.57 ± 1.35*	0.652
Last follow-up	10.29 ± 1.28*	10.28 ± 1.38*	0.960
DH change (mm)	1.63 ± 1.37	1.53 ± 1.12	0.767
CSAC (cm^2^)			
Preoperation	0.65 ± 0.22	0.64 ± 0.19	0.773
Last follow-up	1.70 ± 0.26*	1.78 ± 0.23*	0.253
CSAC change (cm^2^)	1.05 ± 0.35	1.14 ± 0.34	0.326
RLS (%)	92.72 ± 6.39	93.54 ± 5.21	0.599
Fusion rate (%)	89.3	92.9	1.000^a^
Cage subsidence (%)	14.3	17.9	1.000^a^

*LLA, lumbar lordotic angle; LLA, SLA, DH, or CSAC change: postoperative data minus preoperative data; SLA, segmental lordotic angle; DH, disc height; CSAC, cross-sectional area of the spinal canal; RLS, reduction rate of lumbar spondylolisthesis; RLS, reduction rate of lumbar spondylolisthesis; PE-PLIF, percutaneous endoscopic posterior lumbar interbody fusion; Open PLIF, open posterior lumbar interbody fusion.*

^a^ *Results from Fisher’s exact test.*

* *p < 0.05 compared with the preoperative data.*

**Figure 4:**
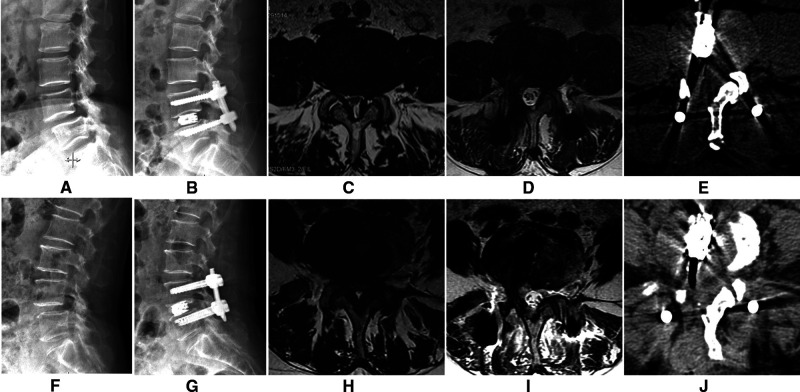
The lateral X-Ray showing L4 spondylolisthesis (**A**), and it was complete reduction after surgery (**B**). A cross-sectional MRI image (**C**) showing lumbar spinal stenosis; the cross-sectional area of the spinal canal significantly improved after surgery (**D**). A cross-sectional CT image (**E**) showing that a part of the lamina, the articular process, and the base of the spinous process are excised to enlarge the spinal canal. The other patient is shown in (**F–J**).

Images of the two cases are shown in Figure 4.

## Discussion

Whether ULBD can be applied in patients with lumbar instability has not been reported, and only a few reports on ULBD for lumbar spondylolisthesis are available (17–19). In a study by Park et al. (17), ULBD achieved satisfactory clinical outcomes for grade I lumbar spondylolisthesis with nerve root symptoms, but foraminal stenosis was a contraindication. In a study by Yoshikane et al. (18), endoscopic ULBD provided favorable outcomes for lumbar spinal stenosis with or without grade I lumbar spondylolisthesis, but 31% of patients with lumbar spondylolisthesis experienced aggravation of their condition. Although a few reports show that ULBD alone can provide positive outcomes, previous studies still support that interbody fusion is an effective method for treating lumbar spondylolisthesis (20).

### Review of Unilateral Laminotomy for Bilateral Decompression

Poletti (2) reported the unilateral laminotomy for bilateral ligamentectomy approach, which involves making a median skin incision and a fascial incision 1 cm laterally, splitting the paraspinal muscles to expose the lamina, excising approximately 8 mm of the ipsilateral superior lamina and a part of the inferior lamina, ligamentum flavum, and the base of the spinous process, excising a part of the contralateral lamina and ligamentum flavum, and performing spinal canal decompression. Spetzger et al. (3, 21) proposed the ULBD approach and provided a detailed surgical technique. The surgical approach is similar to that reported by Poletti; however, ULBD is performed under the assistance of a microscope, and a part of the facet joint is removed to enlarge the spinal canal and lateral recess. Oertel et al. (22) reported a 4-year follow-up study of 133 patients with lumbar spinal stenosis who underwent ULBD. They observed favorable clinical outcomes, concluding that ULBD is a very good surgical method for treating lumbar spinal stenosis. Since 2012, ULBD with a tubular retractor has been used in clinical practice (4, 5). With the incision 0.5–1 cm to the midline, this surgical procedure is basically the same as open ULBD but is believed to reduce intraoperative injury and speed up recovery (4, 5, 23). With advancements in lumbar endoscopic technology, endoscopic ULBD has been widely studied and applied since 2020 (24). The position of the incision is slightly different among reports but is generally 0.5–2 cm from the midline (5–7, 24, 25). Endoscopic ULBD can improve surgical visualization, reduce postoperative low back pain, and shorten postoperative hospital stay (6, 7, 18, 24, 25). Some scholars have reported the utilization of unilateral biportal endoscopic ULBD, with the insertion point being more medial than that in unilateral biportal endoscopic interbody fusion (UBE) to protect facet joints, resulting in positive clinical outcomes (26).

### Endoscopic Lumbar Interbody Fusion and Unilateral Laminotomy for Bilateral Decompression

Endoscopic lumbar interbody fusion includes percutaneous endoscopic transforaminal lumbar interbody fusion (PE-TLIF) (27), UBE (28, 29), and PE-PLIF. We have utilized all of these procedures. The main difference among the approaches is which part of the facet joint is removed. The superior articular process (SAP) is removed to establish a working channel in PE-TLIF (30). The inferior articular process (IAP) and the medial part of the SAP are removed in PE-PLIF (9). The entire articular process is removed in UBE (29). In PE-TLIF, the IAP is preserved, and the working cannula has a larger inclination angle. ULBD cannot be performed during PE-TLIF. In the study of Li et al., if necessary, an additional endoscopic ULBD was performed after PE-LTIF (31). As the working cannula in PE-PLIF is at almost the same position and angle as that in endoscopic ULBD, ULBD can be easily completed during PE-PLIF. Some studies on UBE have also mentioned that ULBD can be performed at the same time, but none of them have been described in detail (28, 32).

### The Advantages of Percutaneous Endoscopic Posterior Lumbar Interbody Fusion with Unilateral Laminotomy for Bilateral Decompression

The working channel for PE-PLIF is located approximately 2 cm paravertebrally, which is almost the same as the classic ULBD surgical approach (9, 11). Thus, ULBD can easily be performed during PE-PLIF. Both interbody fusion and bilateral decompression can be completed at one time to avoid the extra contralateral operation, thereby simplifying procedures and minimizing injury. What differs from the classic ULBD approach is that the initial positioning point is at the junction of the articular process and lamina instead of at the junction of the spinous process and lamina. The procedure for ULBD in PE-PLIF is the same as that previously reported (5, 11). More of the contralateral articular process can be removed without worrying about destroying the stability of the lumbar spine.

This study found that PE-PLIF with ULBD can provide similar surgical efficacy and imaging results as PLIF, but there are some differences, which can be explained as follows. First, the estimated blood loss and postoperative hospitalization of PE-PLIF with ULBD were significantly less than those of PLIF. The drainage tube of open PLIF was usually removed at 3–5 days after surgery. Then, the patients were taken imagings and allowed early ambulation. So, their postoperative stay was 7.3 ± 1.5 days. The patients in the PE-PLIF group underwent the same process, with one difference being the drainage tube was removed 1 day after surgery. The estimated blood loss in our study was intraoperative blood loss plus postoperative drainage volume. This may be the explanation for the significant blood loss. Because the paraspinal muscle and spinous ligament complex were protected in PE-PLIF with ULBD, the postoperative low back pain associated with PE-PLIF with ULBD was significantly lower than that with PLIF surgery. Second, the study found that the improvement in the LLA and SLA was not obvious in either group. Because the SLA and LLA in lumbar spondylolisthesis were larger than those in normal lumbar, the angle may be smaller or slightly larger after spondylolisthesis reduction. Since the entire lamina was removed in PLIF, the CSAC after PLIF was slightly larger than that in the PE-PLIF group. In addition, the RLS was comparable between the two groups, indicating that the degree of soft tissue release during PE-PLIF was sufficient to reduce spondylolisthesis. In conclusion, PE-PLIF with ULBD is effective for the treatment of lumbar spondylolisthesis and lumbar spinal stenosis. The advantages of PE-PLIF with ULBD are reducing postoperative back pain, reducing trauma, and enhancing recovery after surgery. The main disadvantage is the long operative time, which is a common problem for all minimally invasive surgeries. Improvements in both surgical techniques and instruments are needed to reduce the operative time in the future.

### Complications

The incidence of ULBD complications varies among reports. Dural tears are a very common complication, occurring in approximately 6.8%–18% of open surgeries and tubular procedures (22, 33, 34) and 0%–7.2% of endoscopic ULBD procedures (11, 19, 25). The reason for this difference is that clear surgical visualization and careful operation under endoscopy help prevent dural tears in the narrow surgical space where high-speed drills and osteotomes are used. In our study, dural tears occurred in only one patient. Compared with previous studies, we rarely used the osteotome or ultrasonic osteotome instead of burr during the operation, which are more controllable and safer. Studies have reported that the postoperative reoperation rate is approximately 10% due to restenosis of the surgical segment and secondary segmental instability (22). Some surgeons believe that greater articular process preservation during the operation corresponds to a lower risk of postoperative segmental instability (26). However, in ULBD, a part of the facet joint must be excised, and usually, more of the contralateral facet joint needs to be removed (35). Overall, ULBD has low complication rates and satisfactory clinical outcomes, and endoscopic techniques have lower complication rates than open surgery and tubular approaches in most studies. The utilization of endoscopic techniques can improve surgical visualization and reduce the occurrence of complications. The reoperation rate is associated with the selection of indications and how much the facet joint is excised. Since interbody fusion and pedicle screw fixation are performed in PE-PLIF, the risk of reoperation does not need to be considered. In conclusion, PE-PLIF with ULBD is a safe and effective method to expand the indications for ULBD.

### Limitations

This study has some limitations. Although we strictly followed the inclusion and exclusion criteria during case selection, selection bias was inevitable. The sample size was small. Interobserver bias in the measurement of the radiological parameters may have been present.

## Conclusion

The results from this study indicate that ULBD can be successfully performed during PE-PLIF and that the combined procedure is a safe and reliable treatment method for lumbar spondylolisthesis. Compared with open PLIF, PE-PLIF with ULBD is less invasive and leads to enhanced recovery after surgery. Despite the lengthy operation time, we believe that the benefits outweigh the shortcomings.

## Data Availability

The raw data supporting the conclusions of this article will be made available by the authors, without undue reservation.
